# Supplementary material for: Whole‐Mount Acetylcholinesterase Staining Reveals Unique Motor Innervation of the Lamprey Oral Region: With Special Reference to the Evolutionary Origin of the Vertebrate Jaw
Source: J Exp Zool B Mol Dev Evol. 2025 Jul 3;344(6):341–51. doi: 10.1002/jez.b.23316 (PMC12328844; doi:10.1002/jez.b.23316)
Supplement: Supplementary file 1 — Supplement_v1. [file JEZ-344-341-s001.docx]

**Supplementary Information**

**Whole-Mount Acetylcholinesterase Staining Reveals Unique Motor Innervation of the Lamprey Oral Region: With Special Reference to the Evolutionary Origin of the Vertebrate Jaw**

Motoki Tamura^1^ and Daichi G. Suzuki^2*^

^1^Graduate School of Life and Environmental Sciences, University of Tsukuba, Tsukuba, Japan

^2^Institute of Life and Environmental Sciences, University of Tsukuba, Tsukuba, Japan

^*^Correspondence: suzuki.daichi.gp@u.tsukuba.ac.jp

**
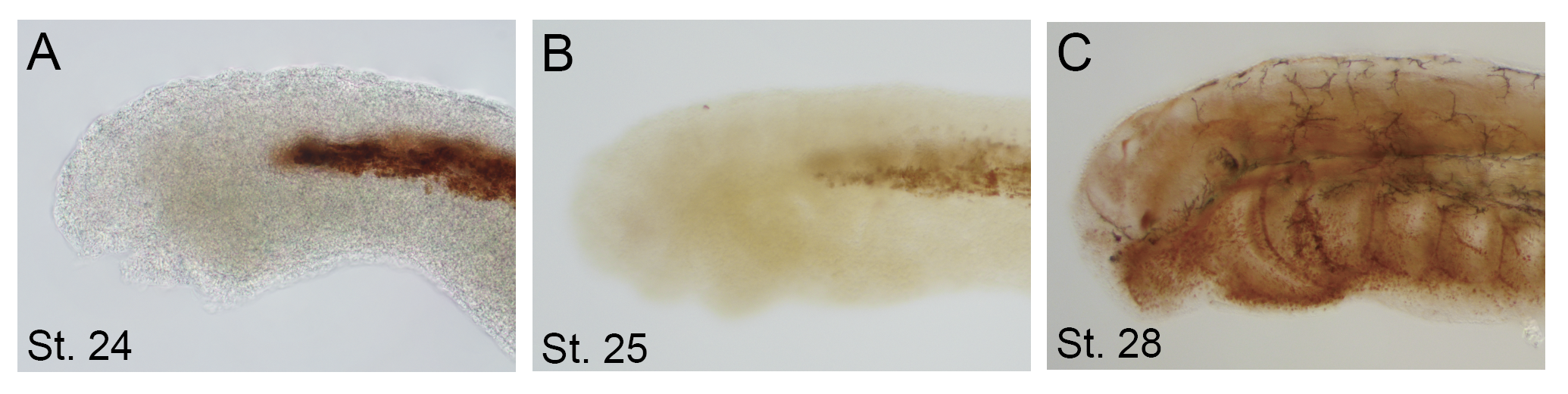
**

**Figure S1. Whole-mount AChE staining of lamprey embryos and prolarva.** (A) St. 24, (B) St. 25, (C) St. 28.
